# Proteomic characterization of adrenal gland embryonic development reveals early initiation of steroid metabolism and reduction of the retinoic acid pathway

**DOI:** 10.1186/s12953-015-0063-8

**Published:** 2015-02-07

**Authors:** Gry H Dihazi, Gerhard A Mueller, Abdul R Asif, Marwa Eltoweissy, Johannes T Wessels, Hassan Dihazi

**Affiliations:** Department of Nephrology and Rheumatology, University Medical Center Goettingen, Georg-August University Goettingen, Robert-Koch-Strasse 40, D-37075 Goettingen, Germany; Department of Clinical Chemistry, Georg-August University Goettingen, Robert-Koch-Strasse 40, D-37075 Goettingen, Germany

**Keywords:** Adrenal gland, Proteomics, Prenatal development

## Abstract

**Background:**

Adrenal glands are essential endocrine organs composed of two embryological distinct tissues. Morphological changes during their development are well described, but less understood with regard to their molecular mechanisms. To identify proteins and pathways, which drive the initial steps of the specification of the endocrine function of the adrenal gland, rat’s adrenal glands were isolated at different embryonic days (E): E14, E16, E18, E19 and postnatal day 1 (P1).

**Results:**

The alteration of the proteome during the stages E16, E19 and P1 was investigated by combining two dimensional gel electrophoresis and mass spectrometric analysis. Out of 594 excised protein spots, 464 spots were identified, resulting in 203 non-redundant proteins. The ontogenic classification of the identified proteins according to their molecular function resulted in 10 different categories, whereas the classification of their biological processes resulted in 19 different groups. This gives an insight into the complex mechanisms underlying adrenal gland development. Interestingly, the expression of retinoic acid pathway proteins was decreased during the development of the adrenal gland, suggesting that this pathway is only important at early stages. On the other hand, key proteins of the cholesterol synthesis increased their expression significantly at E19 revealing the initiation of the endocrine specialization of the adrenal glands.

**Conclusions:**

This study presents the first comprehensive wide proteome analysis of three different stages of embryonic adrenal gland development. The identified proteins, which were expressed in early stages of development, will shed light on the molecular mechanisms underlying embryonic development of the adrenal gland.

**Electronic supplementary material:**

The online version of this article (doi:10.1186/s12953-015-0063-8) contains supplementary material, which is available to authorized users.

## Background

Adrenal glands (AGs) are complex endocrine organs, which are vitally necessary. Each gland is composed of two embryological distinct tissues, the mesodermal cortex and the medulla, arising from the neural crest ectoderm. The adrenal medulla produces catecholamine, such as epinephrine (adrenaline) [1,2], in the classic immediate stress response (fight or flight), whereas the adrenal cortex regulates the long-term stress response by modulating salt, pH, and glucose homeostasis through the synthesis of corticosteroids, such as cortisol [3,4].

The development of the adrenal gland begins with the migration of neural crest cells toward the coelomic cavity wall and forms a thickening at the medial side of the fetal cortex anlage of the adrenal gland. When the fetal cortex surrounds these cells, they start to differentiate into catecholamine secreting chromaffine cells of the adrenal medulla. Later, further mesenchymal cells detach from the mesothelium and surround the fetal cortex, building the permanent cortex [5,6].

The adrenal cortex, the outer 90% of the gland, synthesizes corticosteroid hormones from cholesterol. Its major secretions are cortisol, an important metabolic hormone, androgens such as androstenedione and dehydroepiandrostenedione (DHEA), and aldosterone, a hormone regulating water and electrolyte concentrations [5,6]. The adrenal medulla, the inner 10% of the gland is the main source of the catecholamines epinephrine (adrenaline) and norepinephrine (noradrenaline).

The adrenal gland is an organ, which undergoes major postnatal developmental modifications [7-9]. Recently, a differential proteomic analysis of adrenal gland during postnatal development focusing on regulated proteins, which are involved in steroidogenesis, cell proliferation, and cholesterol synthesis [10] was published.

The underlying molecular mechanisms of the prenatal development of the adrenal gland are still not completely understood. To unravel the protein expression changes that accompany the AG embryonic development, two main approaches might be considered: the analysis of the transcriptome and proteome investigation. Transcriptomic analysis is a powerful method, widely used for comparison of gene expression of different biological samples. Nonetheless, mRNA translation might be influenced by many factors, which can have a profound impact on the amount of protein synthesized. Additionally, post-transcriptional and post-translational events, which play important roles in embryonic development, increase the diversity of proteins that can be synthesized from a fixed number of genes. Moreover, gene expression changes, which result in alteration in mRNA level, do not necessarily result in protein expression or activity modification. Proteome investigation might help to overcome the limitations of the transcriptomic analysis and deliver important insights into the molecular mechanisms of AG development on the proteome level. For these reasons we decided to use proteomics methods to investigate changes in the protein expression during AG embryonic development. We performed a comparative proteomic analysis of two prenatal stages (embryonic day 16 (E16) and 19 (E19)) as well as of newborn rats (postnatal day 1 (P1)). Here we report some of the molecular modifications identified, and discuss the subjacent functions of the differentially expressed proteins.

## Results

### Preparation and HE staining of the adrenal glands

**Figure 1:**
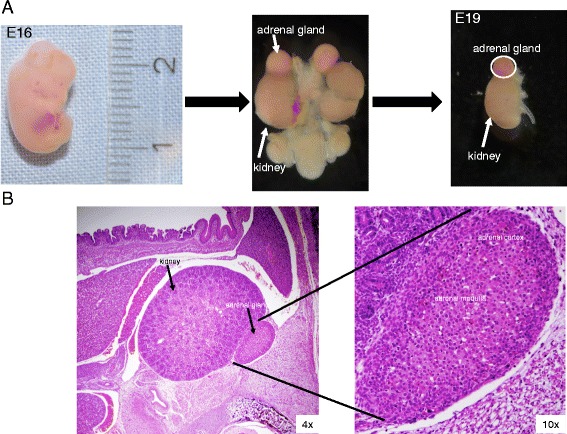
**Adrenal gland preparation. A**: Embryos were removed from the mother at different days of development. The adrenal glands were excised from the embryos as well as from the newborn rats (P1). **B**: HE staining of a paraffin section of a rat embryo at E19 showing the kidney and the adrenal gland, enclosing the adrenal cortex and the adrenal medulla.

To identify and characterize proteins and pathways involved in the development of the adrenal gland, rats were obtained at different stages of embryonic (E14, E16, E18 and E19) and neonatal (P1) development and the AGs were obtained (Figure 1A). The HE staining of the adrenal gland from E19 embryos showed the two distinct tissues of this endocrine organ, the mesodermal cortex, which is the zone of steroid-synthesizing cells and the medulla, which consists of cells arising from the neural crest ectoderm and is responsible for synthesis and secretion of catecholamines (Figure 1B).

### Mapping of embryonic adrenal glands proteome

Proteins were extracted and purified from adrenal glands homogenates, and separated by 2-D gel electrophoresis as described in material and methods. The 2-DE analysis was performed using immobilized pI gradients (IPG) (pI 5–8) in the first dimension, and SDS-PAGE in the second dimension. To investigate protein pattern differences between E16 and E19/P1, three independent 2-DE images of each protein extract from three independent biological replicates were selected for comparative and statistical analyses. We assessed the degree of 2-DE technical variation by determining the coefficient of variation (CV) for all spot volumes on a gel for all technical replicates. Protein spot volumes were determined for all matched spots in an experiment set using Delt2D software. We observed only minimal differences in CV values from replicate gels from the same embryonic stage, more than 85% of the matched spots have a CV% < 20%. To investigate the biological variation that can result from the use of different pregnant female animals for AG isolation, CV% was determined for AG 2-DE from same embryonic stage isolated from different pregnant female. Depending on the embryonic stage, between 940 and 1150 protein spots were detected on 2-DE gels. The CV% values were below 10% for 25–31% of the spots and below 20% for 75–86% of the spots.

**Figure 2:**
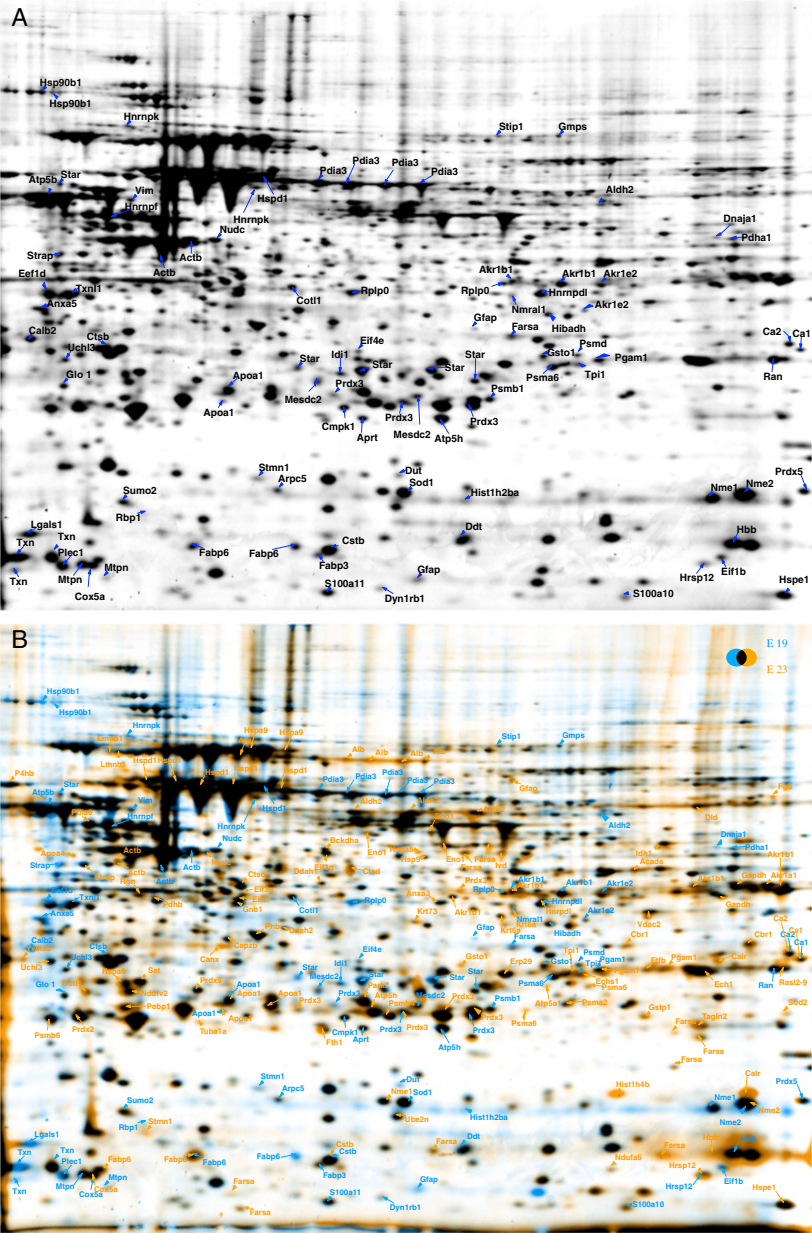
**2-DE gel of the adrenal gland. A**: 2-DE reference map of proteins extracted from the adrenal gland of an E19 embryo. 150 μg proteins were loaded on an 11-cm IPG strip with a linear pH gradient pI 5–8 for IEF, 12% SDS-polyacrylamide gels were used for the SDS-PAGE. Proteins were stained with Flamingo fluorescent gel stain. Identified spots were assigned a gene name. **B**: An overlay of the 2D gel images of the adrenal glands of E19 embryo and newborn. The identified spots are labeled with the gene names.

The spots detected by image analysis in the Flamingo-stained gels are presented in Figure 2A for E19 and in Additional file 1: Figure S1 for E16 (A) and P1 (B). The gels were analyzed by Delta 2D Version 4.3 (Decodon, Braunschweig). The 2-DE gel images of the different stages were overlaid and carefully compared. Evaluation of the 2-DE maps of the three stages revealed high similarity (>95% spots overlapping) in the protein pattern between the late embryonic (E19) and newborn (P1) stages, whereas the adrenal glands from the early embryonic stage (E16) showed different protein patterns than E19 and P1 (Figure 2B and Additional file 1: Figure S1C, D). To obtain an overview of the existing proteins in the AG at the different stages, more than 500 protein spots were excised from the 2-DE gels and further processed for identification. Protein spots, which were present at all three investigated AG stages, were only excised for identification from one of the gels.

**Figure 3:**
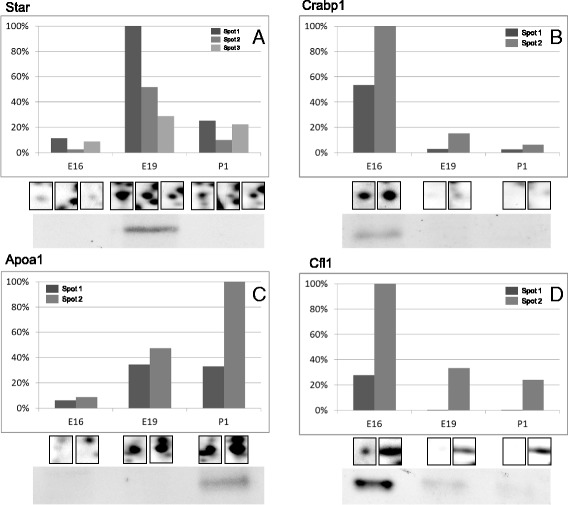
**Temporal change in the expression of selected proteins.** Graphs represent enlargement of the gel regions of interest showing protein spots found to be differentially expressed in AG from different embryonic stages. The protein expression quantification for selected proteins is given in form of bar diagrams. On the y-axis the relative intensity of spot is given on the x-axis the corresponding embryonic stage from which the AGs were collected. Results are given as the means ± SD of the percentage volume of spot as quantified by Delta 2D software. All the proteins shown, present significant expression changes during AG development (P < 0.05). Three different spots (Spot 1, 2 and 3) were identified as Star **(A)**, two spots (Spot 1 and 2) were identified as Crabp1 **(B)**, two spots (Spot 1 and 2) were identified as Apoa1 **(C)**, and two spots (Spot 1 and 2) were identified as Cfl1 **(D)**. Western blot analyses confirming the protein expression changes are provided.

The excised spots were in-gel digested with trypsin and processed for mass spectrometric analysis (MS/MS). Proteins were identified by the sequence databases search using Mascot. A total of 464 protein spots were identified resulting in 203 non-redundant proteins (Additional file 2: Table S1). Some of the proteins were identified in multiple spots, as shown in Figure 3.

**Figure 4:**
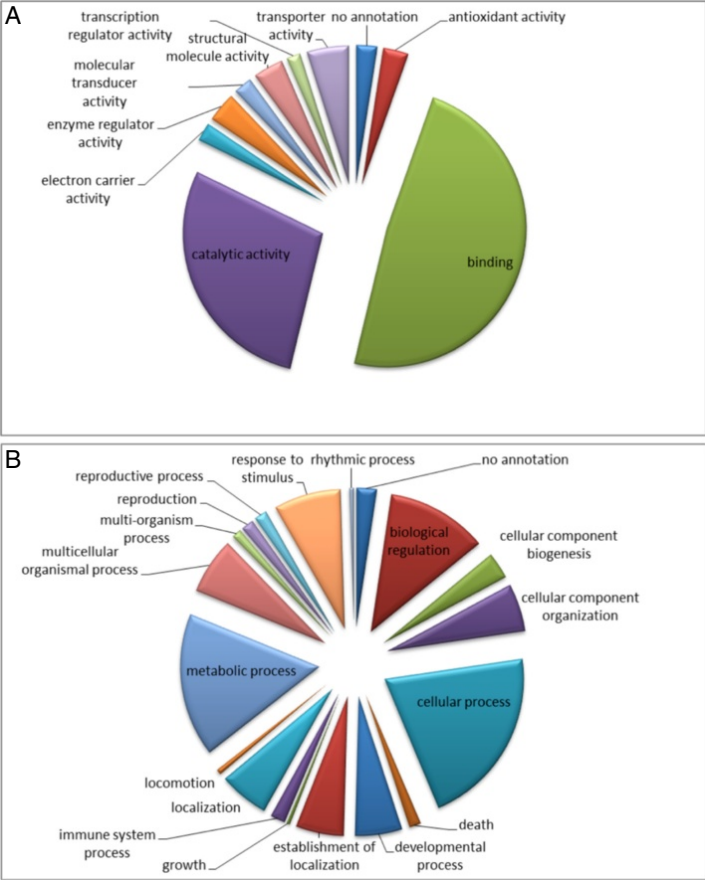
**Pie charts of the obtained categories after analysis with DAVID bioinformatics. A**: Classification of all identified proteins in the AG according to their molecular function using David Bioinformatics. Most of the proteins were found to be involved in binding functions or catalytic activity. **B**: Distribution of all identified proteins according to their involvement in biological processes.

To gain more information on the biological mechanisms of the identified proteins in embryonic AGs, we combined DAVID bioinformatics with information on the putative function of the protein found in the UniProt and GenBank databases. Thereby, we were able to annotate all 203 gene products. An analysis of the molecular function of the identified proteins based on the Gene Ontology (GO) terms allowed the classification of the proteins into ten different categories (Figure 4A); approximately half of these proteins were classified as binding proteins (48%, 180 proteins) and about one-fourth were involved in catalytic activities (28%, 106 proteins). The rest of the proteins were classified in other categories with less than 5% (20 proteins) per group e.g. transcription regulation, enzyme activity and antioxidant activity. The ontological classification of the identified proteins according to their postulated involvement in biological processes resulted in 19 different categories revealing the complexity of mechanisms that drives embryonic development of adrenal glands (Figure 4B).

### Protein expression changes during embryonic development: alteration in cholesterol metabolism and retinoic acid pathway proteins

**Figure 5:**
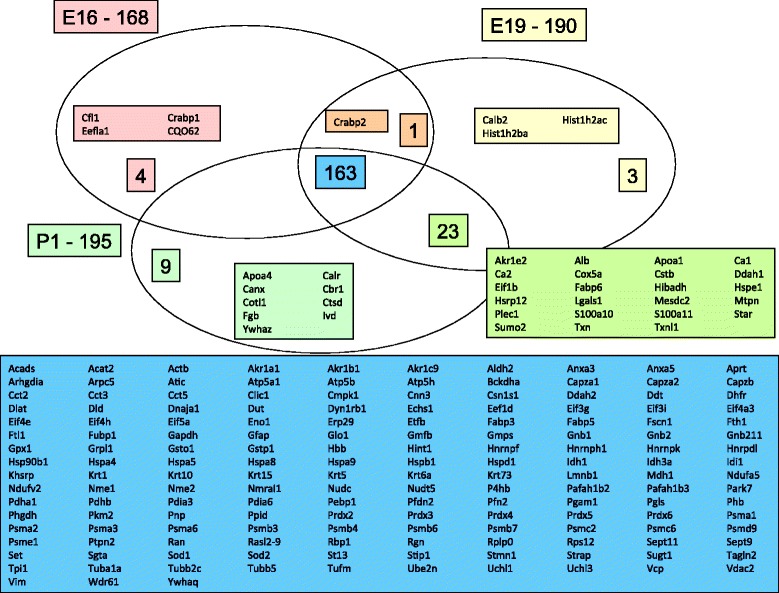
**Venn diagram of the distribution of the identified proteins in the adrenal gland at the different days of development.** At day E16 a total of 168 proteins were identified, of which 4 were only present at day E16, 1 was present at day E16 and E19 and 163 were present at all three examined developmental days. At day E19 a total of 190 proteins were identified, of which three were only present on the given stage, whereas 23 were present at day E19 and P1. Of the 195 proteins, which were identified in the newborn rat, 9 were only present at this embryonic stage.

A total of 168 non-redundant proteins were identified in E16, 190 proteins in E19, and 195 proteins in P1 (Figure 5). Comparative analysis of the protein expression with regards to the embryonic stage showed that 163 proteins were present in all three stages, whereas the other proteins were uniquely found in one or two stages (Figure 5). Several proteins, which were identified in all stages, showed significant expression regulation between E16 and P1. An examination of all regulated proteins revealed 48 (31 non-redundant) proteins for which expression was increased in P1 (Table 1) and 48 proteins (36 non-redundant) for which expression was decreased in stage P1 (Table 2).

**Table 1:** **Proteins, which expression increased throughout the embryonic development of the adrenal gland**

**Increased proteins**	**E16/P1**	**p-value**	**Increased proteins**	**E16/P1**	**p-value**
Actb	0.25	0.0013	Hnrnpk	0.21	0.0381
Actb	0.29	0.0062	Hnrnpk	0.33	0.0410
Akr1e2	0.31	0.0105	Hrsp12	0.04	0.0013
Alb	0.01	0.0141	Hspa9	0.40	0.0044
Alb	0.06	0.0236	Hspa9	0.40	0.0182
Aldh2	0.30	0.0394	Hspa9	0.41	0.0381
Aldh2	0.52	0.0103	Hspd1	0.40	0.0416
Apoa1	0.06	0.0416	Hspe1	0.04	0.0249
Apoa1	0.17	0.0495	Krt73	0.50	0.0007
Apoa1	0.17	0.0210	Mesdc2	0.19	0.0156
Apoa4	>0.01	0.0150	Pebp1	0.31	0.0031
Atp5a1	0.39	0.0021	Pebp1	0.43	0.0461
Ca2	0.15	0.0043	Phb	0.46	0.0037
Calr	0.04	0.0021	Prdx3	0.01	0.0441
Cmpk1	0.43	0.0071	Prdx3	0.35	0.0134
Cotl1	0.16	0.0092	Prdx3	0.12	0.0023
Cox5a	0.19	0.0115	Prdx3	0.17	0.0372
Cox5a	0.22	0.0003	Prdx3	0.26	0.0501
Ctsd	0.20	0.0046	Star	0.42	0.0019
Eno1	0.17	0.0064	Star	0.52	0.0021
Fabp3	0.42	0.0223	Tpi1	0.46	0.0291
Fabp6	0.14	0.0069	Tpi1	0.33	0.0063
Fabp6	0.30	0.0022	Tuba1a	>0.01	0.0002
Hibadh	0.35	0.0070	Txn	0.10	0.0012

**Table 2:** **Proteins, which expression decreased throughout the embryonic development of the adrenal gland**

**Decreased proteins**	**E16/P1**	**p-value**	**Decreased proteins**	**E16/P1**	**p-value**
Akr1b1	6.4	0.0002	Khsrp	2.6	0.0268
Anxa3	2.3	0.0012	Khsrp	3.1	0.0052
Arpc5	2.0	0.0405	Krt6a	2.8	0.0306
Cct3	3.9	0.0052	Krt6a	3.3	0.0188
Cfl1	3.7	0.0061	Krt73	4.1	0.0471
Cfl1	<10	0.0012	Lmnb1	2.2	0.0089
Clic1	2.1	0.0026	Pdia3	2.4	0.0165
Crabp1	<10	0.0003	Pdia3	2.5	0.00401
Crabp1	<10	0.0019	Pdia3	3.6	0.00171
Crabp2	7.3	0.0001	Pdia3	2.0	0.0413
Dhfr	2.2	0.0072	Phgdh	2.4	0.0021
Eef1a1	<10	0.0028	Pkm2	2.3	0.0346
Fscn1	6.5	0.00174	Prdx5	2.6	0.0059
Fscn1	7.3	0.0083	Rbp1	3.8	0.0002
Fubp1	3.1	0.0373	Sept11	2.7	0.0073
Gfap	2.9	0.0367	Stip1	2.1	0.0144
Gmps	3.4	0.0210	Stip1	2.3	0.0185
Gnb1	2.1	0.0209	Stmn1	2.0	0.0027
Gnb211	3.6	0.0196	Stmn1	3.7	0.0003
Hnrnph1	2.1	0.0303	Tubb5	<10	0.0001
Hnrnph1	2.7	0.0415	Ube2n	2.2	0.0221
Hnrpdl	2.2	0.0046	Vcp	2.5	0.0031
Hsp90b1	4.4	0.0275	Vim	2.5	0.0001
Hspa4	3.7	0.0342	Vim	3.7	0.0012

**Figure 6:**
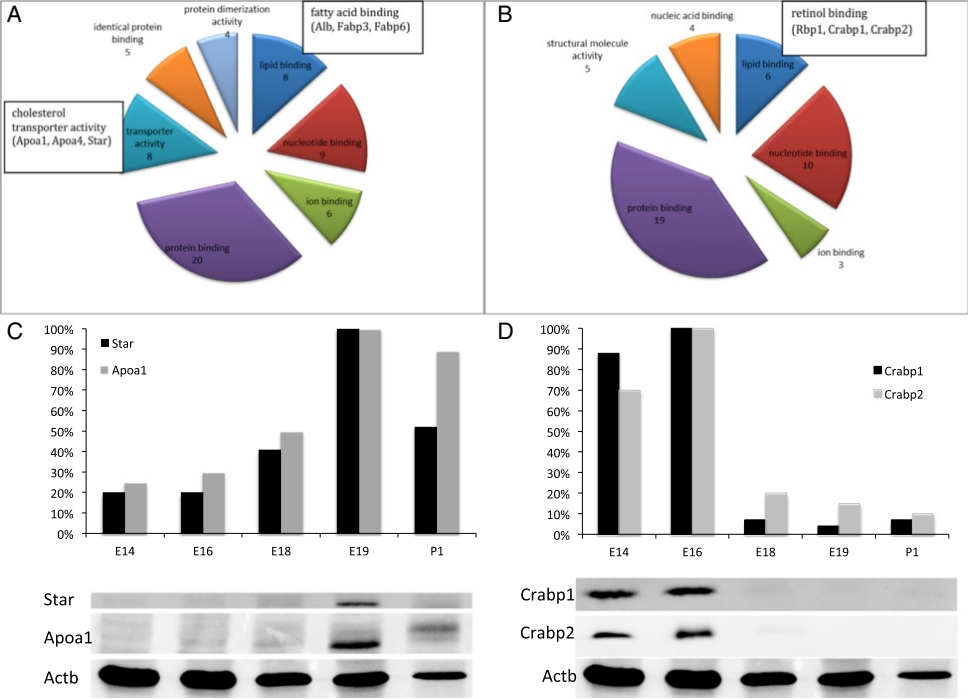
**Pie charts and diagrams of regulated proteins. A**: Distribution of the proteins, which were increased during the development of the adrenal gland, according to their molecular function. **B**: The diagram shows the protein intensity based on Western blot analysis. **C**: Distribution of the proteins, which were decreased during the development of the adrenal gland, according to their molecular function. **D**: The diagram shows the protein intensity based on Western blot analysis.

**Figure 7:**
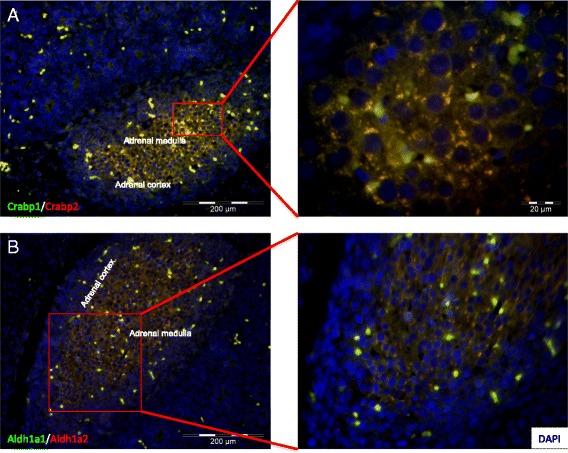
**Immunohistological fluorescence staining of the adrenal gland at day E19.** The fluorescence staining shows a higher expression of the proteins Crabp1 and Crabp2 **(A)** and Aldh1a1 and Aldh1a2 **(B)** in the adrenal medulla than in the adrenal cortex. The slides were analyzed on an immunofluorescence Zeiss Axiophot microscope (Carl Zeiss, Jena, Germany) using the AnalySIS software (Soft Imaging Systems, Leinfelden, Germany).

The ontological classification of proteins for which expression was significantly altered in AGs during embryonic development, was performed according to their molecular function (Figure 6A, 6B). Although the regulated proteins were divided into two groups; proteins for which expression was increased (Table 1) and proteins for which expression was decreased (Table 2), they partly belong to the same categories of molecular function. Six proteins for which expression was decreased and eight for which expression was increased are involved in lipid binding. Three of the proteins, which expression was increased, are involved in fatty acid binding (Alb, Fabp3, and Fabp6) (Figure 6A), whereas three of the proteins for which expression was decreased, are involved in retinol binding (Rbp1, Crabp1, and Crabp2) (Figure 6B). The expression of some of these proteins was confirmed by Western blot analysis (6C, D). The expression of proteins, which are involved in transporter activity, especially cholesterol transporter activity, was increased in the adrenal gland during embryonic development (Apoa1, Apoa4, and Star) (Figure 6A, 6C). Among these proteins, the expression of Star (steroidogenic acute regulatory protein, mitochondrial) an essential cholesterol transporting protein, which is expressed exclusively in adrenal cortex, was significantly affected. Star is a protein, which plays a key role in steroid hormone synthesis by enhancing the metabolism of cholesterol into pregnenolone. Star was barely expressed in early stage of adrenal gland development (E16), whereas in later stage (E19) the expression increased significantly. The protein seems to be posttranslationally modified as evidenced by different spots with similar molecular weights but different pIs (Additional file 3: Figure S2). The proteins involved in the retinoic acid pathway, especially Crabp1, Crapb2, Aldh1a1 and Aldh1a2 were detected in the adrenal medulla shown by immunofluorescence staining of histological sections (Figure 7A, 7B).

## Discussion

To date, the growth and function of the adrenal gland have been predominantly investigated in the postnatal and adult stages [10]. Detailed studies of the molecular mechanisms involved in embryonic development of the AGs are still missing. In order to elucidate the molecular mechanisms associated with the embryonic development of the AG, we investigated the proteome changes between early and late embryonic stage of development of the AGs. The data obtained from this study suggest developmental stage specific pathway activation. Taking the biological and functional classification of the proteins, which were differentially expressed during the development into account, we could observe, that expression of the proteins involved in biological processes like response to stress (Ube2n, Gfap, Vcp, Pkm2, Akr1b1, Prdx5, Hspa4), cell cycle process (Phgdh, Stmn1, Tubb5), transport (Khsrp, Crabp1, Crabp2, Clic1, Cfl1, Rbp1, Vcp), gene expression (Fubp1, Hnrpdl, Khsrp), cell differentiation (Cfl1, Phgdh, Stmn1), and embryonic development (Crabp2, Cfl1, Phgdh) decreases in the course of development (Table 2). In contrast, the expression of proteins, which are involved in lipid metabolic process (Apoa4, Tpi1, Ech1, Apoa1, Star, Prdx6, Fabp3, Atp5a1, Fabp6), steroid metabolic process (Apoa1, Apoa4, Star, Fabp6), response to stress (Star, Phb, Prdx3, Cotl1, Apoa4, Apoa1, Alb, Aldh2, Pebp1, Ctsd, Hspe1, Hspd1), cell differentiation (Actb, Apoa1, Apoa4, Star, Pebp1, Prdx3, Calr), neurogenesis (Actb, Apoa1, Apoa4, Calr, Star), and neuron development/differentiation (Actb, Apoa1, Apoa4) increases in the course of development (Table 1).

The proteomics data revealed interesting aspects in the AG embryonic development. First, the expression of key proteins of the RA-pathway decreases in the course of AG development suggesting that this pathway is only important in early stages of development. Second, proteins, which are important in the steroid biosynthetic process or sterol transporter activity, like Apoa1, Apoa4, Star, Fabp3 or Fabp6 were hardly expressed in early stage, whereas their expression increased in the course of development of the adrenal gland. Star (Steroidogenic acute regulatory protein, mitochondrial) stimulates the regulated production of steroid hormones in the adrenal cortex and gonads by facilitating the delivery of cholesterol to the inner mitochondrial membrane [11], which is the rate-limiting step in the production of steroid hormones [12,13]. Production of steroid hormones is one of the main functions of the adrenal cortex. Whereas Star was not detected in the early embryonic stage (E16), four spots were identified as Star in later stages (E19, P1). These spots may represent four different isoforms of the protein. Three of these were verified by 2-D Western blot analysis. Apoa1, Apoa4 and Fabp6 appeared in late stage of AG development, Apoa1 participates in the reverse transport of cholesterol from tissues to the liver for excretion by promoting cholesterol efflux from tissues and by acting as a cofactor for the lecithin cholesterol acyltransferase (LCAT). Apoa4 may have a role in chylomicrons and VLDL secretion and catabolism. It is also required for efficient activation of lipoprotein lipase by ApoC-II, which is a potent activator of LCAT. Apoa4 is a major component of HDL and chylomicrons. The expression of proteins involved in steroid metabolic process and cholesterol synthesis increased significantly in later stages suggesting that the embryonic development of the adrenal gland is accompanied by coordinated metabolic modifications that facilitate the functional role of the gland.

Another important aspect of the embryonic development of AGs seems to be the slowdown of the RA-pathway as revealed by the down-regulation of the key proteins of this pathway in the late prenatal stage. Proteins, which are involved in retinoic acid pathway, like cellular retinoic acid binding proteins (Crabp1, Crabp2) and the retinol binding protein (Rbp1), were found to be expressed in early stages in the AG, but could not be detected in late embryonic stages and new born rats. Rbp1 is binding and transporting retinol in the cell. Once in the cell, retinol is converted into retinoic acid. Crabp1 and Crabp2 are important for the transport of retinoic acid from the cytosol to the nucleus, where it serves as a ligand for nuclear retinoic acid receptors (RARs) that directly regulate gene transcription [14-16] especially of genes that modulate the overall development of the embryo. The down-regulation of the RA-pathway proteins during embryonic development suggests the restricted importance of this pathway in the AG development.

There are several limitations arising from the 2-D electrophoresis as a separation method. The reproducibility of the protein patterns is an issue when using this technique and the range of molecular weight that is resolvable by the method is limited, affecting the detection of very large and very small proteins. Hydrophobic, highly acidic or highly basic proteins are poorly detected resulting in less information on proteome. Moreover, the fact that AGs are constituted from two types of tissue, it might be more informative to resolve the proteome of the two parts separately. This requires additional methods for cell separation or tissue microdissection, which will result in severely reduced amount of usable sample. This will require an even higher number of embryos to be involved in the study and/or more sensitive analytical methods.

## Conclusions

In summary, this study provides preliminary proteomic maps of AGs embryonic development and highlights the embryonic stage specific pathway modulation. The RA-pathway seems to be important at the initial steps of the AG development, whereas the molecular changes in later stages revealed an increased importance of the regulation of steroid hormones synthesis as a first step towards the endocrine function of this organ. Additional investigations are still needed to elucidate the specific role of the single proteins in the AG development and maturation.

## Methods

### Animals

Wistar Han rats were kept under 12:12 h cycle of light with *ad libitum* access to food and drink. Pregnant rat females were used to collect the embryos at different embryonic stages: embryonic day 14 (E14), 16 (E16) 18 (E18) and 19 (E19), and newborn (P1). The adrenal glands (AG) were dissected from these embryos as well as from neonatal pups. To prepare the AG protein extracts, 60 AGs were used in the E14, 90 in E16, 50 in E18, 60 in the E19 extracts, and 30 AGs were used for the P1 extract. All experimental procedures were performed according to the German animal care and ethics legislation (NIH standards) and were approved by the local government authorities.

### Protein extraction

The protein extraction for 2-D gel electrophoresis was performed as described previously [17]. A single AG, especially from embryonic stage E14 and E16 (200 – 300 μm diameter), will not deliver enough protein for 2-DE analysis. Embryos from the same pregnant rat females have the same genetic background and the AGs from these embryos can be pooled together for proteomic analyses. The AGs from embryos at the same embryonic stage and from the same female (between 14–17 embryos) were pooled, the lysis buffer (9.5 M urea, 2% CHAPS (w/v), 2% ampholytes (w/v), 1% DTT) was added and the samples were vortexed. Thereafter, the samples were incubated for 30 min at 4°C. For removing the cell debris, centrifugation was carried out for 30 min at 13,000 ×g and 4°C. The supernatant was recentrifuged at 13,000 ×g and 4°C for an additional 30 min to get maximal purity. The pellet was discarded, and the resulting samples were used immediately or stored at −80°C until use.

### Protein precipitation

To reduce the salt contamination and to enrich the proteins, methanol-chloroform-precipitation according to Wessel and Flugge [18] was performed. Briefly, 0.4 ml methanol (100%) was added to 0.1 ml protein sample and mixed together. 0.1 ml chloroform was added to the sample and the mixture was vortexed. Subsequently 0.3 ml water was added and the solution was vortexed and centrifuged at 13,000 ×g for 1 min. The aqueous layer was removed, and another 0.4 ml methanol (100%) was added to the rest of the chloroform and the interphase with the precipitated proteins. The sample was mixed and centrifuged for 2 min at 13,000 ×g and the supernatant was removed. The pellet was vacuum dried and dissolved in lysis buffer.

Total protein concentration was determined using Bio-Rad protein assay (Bio-Rad, Hercules, CA, USA) according to Bradford [19]. Bovine serum albumin (Sigma, Steinheim, Germany) was used as standard.

### 2D gel electrophoresis (2-DE)

To assure for high data quality 2-DE, five biological replicates consisting of five pregnant rats (each 14–17 embryos) for every embryonic stage were prepared. For embryonic AGs isolated from embryos collected from the same mother at least three independent experimental replicates from each embryonic stage as well as from newborn pups were performed. IPG strips (11 cm, pI 5–8) were passively rehydrated for 12 h in 185 μl rehydration buffer (8 M urea, 1% CHAPS, 1% DTT, 0.2% ampholytes, and a trace of bromophenol blue) containing 150 μg protein. The IEF step was performed on the PROTEAN^(R)^ IEF Cell (Bio-Rad, Hercules, CA, USA). Temperature-controlled at 20°C, the voltage was set to 500 V for 1 h, increased to 1000 V for 1 h, 2000 V for 1 h and left at 8000 V until a total of 50000 Vhours was reached. Prior to SDS-PAGE, the IPG strips were reduced for 20 min at room temperature in SDS equilibration buffer containing 6 M urea, 30% glycerol, 2% SDS 0.05 M Tris–HCl, and 2% DTT on a rocking table. The strips were subsequently alkylated in the same solution with 2.5% iodoacetamide substituted for DTT, and a trace of bromophenol blue. For the SDS-PAGE 12% BisTris Criterion precast gels (Bio-Rad, Hercules, CA, USA) were used according to manufacturer’s instructions. The gels were run at 150 V for 10 min followed by 200 V until the bromophenol blue dye front had reached the bottom of the gel.

### Gel staining

For image analysis, 2-DE gels were fixed in a solution containing 50% methanol and 12% acetic acid overnight and fluorescent stained with Flamingo fluorescent gel stain (Bio-Rad, Hercules, CA, USA) for minimum 5 h. Thereafter, gels were scanned at 50 μm resolution on a Fuji FLA-5100 scanner using the Image Reader Software (Fuji). The digitalized images were analyzed using Delta 2D 4.3 (Decodon, Braunschweig, Germany). For protein identification, 2-DE gels were additionally stained with colloidal Coomassie blue, Roti-Blue (Roth, Karlsruhe, Germany) overnight.

### Protein identification

Manually excised gel plugs were digested as described previously [20]. After digestion the supernatant was removed and saved, and the additional peptides were extracted with different acetonitrile/trifluoroacetic acid ratio under sonication. All supernatants were pooled together, dried in a vacuum centrifuge, and dissolved in 0.1% formic acid. The mass spectrometric sequencing was performed as described previously [21]. Briefly, the tryptic peptides were subjected to mass spectrometric sequencing using a Q-TOF Ultima Global mass spectrometer (Micromass, Manchester, UK).

Processed data were searched against MSDB and Swiss-Prot databases through Mascot search engine using a peptide mass tolerance of 50 ppm (parts per million) and fragment tolerance of 100 mmu (millimass unit). Protein identifications with at least two peptides sequenced were considered significant.

### Bioinformatics

The classification of the identified proteins according to their main known/postulated functions was carried out using DAVID bioinformatics [22,23]. This classification together with the official gene symbol (given in Additional file 2: Table S1) was used to investigate and categorize the gene ontology (GO)-annotations (biological processes and molecular functions).

### Histochemistry

Paraffin embedded sections were first heated in an oven at 65°C for 1 h before undergoing several series of washes of xylene and ethanol to deparaffinize/rehydrate. After deparaffinization/rehydration, the slides were immersed in Hematoxylin solution (Merck) for 3 min. The slides were rinsed with water, and counterstained with 0.5% Eosin G-solution (Merck) for 5 min. After 30 s of rinsing in water, the slides were dehydrated in series of increasing ethanol and xylene concentrations. The coverslips were mounted on the slides with Entellan Neu mounting medium (Merck).

### Immunohistochemistry

To monitor the expression of the selected proteins in the adrenal gland, indirect immunofluorescence staining of proteins of interest was performed. The deparaffinization of the sections was carried out as described above, thereafter the slides were immersed into a staining dish containing Antigen retrieval solution (18 mM citric acid, 82 mM sodium citrate, pH 6.0) and warmed in a food steamer for 25 min. The slides were allowed to cool down for 20 min before being washed with TBST for 5 min. Inactivation of endogenous peroxidase was performed with 3% hydrogen peroxide for 10 min at 37°C. After three successive washing steps with TBST, sections were blocked with 10% goat serum for 60 min. The incubation with the primary antibodies was carried out overnight at 4°C in a humidified chamber. Molecular Probes Alexa Fluor 647 goat anti-mouse IgG antibody or Alexa Fluor 647 goat anti-rabbit IgG (1:200) were used as secondary antibodies. The incubation was performed at room temperature for 60 min in the dark. The coverslips were mounted on the slides using fluorescence mounting medium with DAPI (Vector Laboratories, Inc., Burlingame, USA). Slides were analyzed on a Zeiss Axiophot microscope (Carl Zeiss, Jena, Germany) using the AnalySIS software (Soft Imaging Systems, Leinfelden, Germany).

### Western blot analysis

The validation of the 2-DE data was carried out using Western blot analysis. To assure for the reproducibility of the Western blot analysis, at least three biological and experimental replicates were performed. 40 μg proteins were separated by SDS-PAGE and transferred to Hybond ECL nitrocellulose membrane (GE Healthcare). Immunodetection was performed according to Towbin et al. [24]. Briefly, membranes were blocked in 5% milk for 2 h at room temperature, followed by overnight incubation at 4°C with diluted specific primary antibody. Mouse monoclonal anti-CRABP1 (1:1000) (abcam), rabbit anti-Cofilin (1:1000) (sigma), mouse monoclonal anti-StAR (1:250) (abcam) and mouse monoclonal anti-ß-actin (1:5000) (sigma) were used as primary antibodies. Molecular Probes Alexa Fluor 647 goat anti-mouse IgG antibody or Alexa Fluor 647 goat anti-rabbit IgG (1:2000) were used as secondary antibodies. Before imaging, the blots were dried in the dark. The blot membranes were scanned at 50 μm resolution on a Fuji FLA-5100 scanner (Fuji Photo) with single laser-emitting excitation light at 635 nm.

### 2D Western blot analysis

150 μg proteins were separated by isoelectric focusing and SDS-PAGE as described above and transferred to Hybond ECL nitrocellulose membrane (GE Healthcare). Immunodetection was performed as described above. Mouse monoclonal anti-StAR (1:250) (abcam) was used as primary antibodies. Molecular Probes Alexa Fluor 647 goat anti-mouse IgG antibody (1:2000) was used as secondary antibodies. Before imaging, the blots were dried in the dark and scanned as described above.

### Statistical analysis

For 2-DE the digitalized images were analyzed; spot matching across gels and normalization were performed using Delta2D 4.3 (Decodon, Braunschweig, Germany). Delta2D computes a 'spot quality' value for every detected spot. This value shows how closely a spot represents the 'ideal' 3D Gaussian bell shape. Based on average spot volume ratio, spots whose relative expression is changed at least 2-fold (increase or decrease) between the compared samples were considered to be significant. To analyze the significance of protein regulation, Student's t-test was performed, and statistical significance was assumed for P values less than 0.01.

All blots were quantified using the ImageJ software. For comparison between two measurements (in the same group) paired t-test was used. Unpaired t-test (for comparing 2 groups) or one-way ANOVA (comparing 3 or more groups) were used. The data were compiled with the software package GraphPad Prism, version 4. The software was used for graphical presentation and statistical analysis. Results are presented as the mean ± SD of at least three independent experiments. Differences were considered statistically significant when p < 0.05.

## Additional files

Additional file 1: Figure S1.
**A**: 2D gel of the proteins in the adrenal gland of an E16 embryo and **B**: of a newborn rat. The identified protein spots are labeled with the gene names on the gel. **C**: An overlay of the 2D gels of the adrenal glands of E16 and E19 embryos. **D**: An overlay of the 2D gels of the adrenal glands of E16 embryo and new born. The identified spots are labeled with the gene names. E: HE staining of a paraffin section of adrenal gland and kidney at E19. Additional file 2: Table S1. List of proteins of non-redundant proteins identified from adrenal glands in all three analysed embryonic stages. Gene name, calculated isoelectric point (CIP) and MS/MS information are given. Additionally protein nominal mass and the number of peptides that were sequenced through MS/MS are also given. Additional file 3: Figure S2. 2D Western blot of Star in the adrenal gland at E19. Three different spots with the same molecular mass, but with different pI were observed.

## Abbreviations

AG: Adrenal gland
E16: Embryonic stage day 16
E19: Embryonic stage day 19
P1: Postnatal day 1
2-DE: Two-dimensional gel electrophoresis
CV: Coefficient of variation
GO: Gene ontology
