# Correction to: Proteomic characterization of adrenal gland embryonic development reveals early initiation of steroid metabolism and reduction of the retinoic acid pathway

**DOI:** 10.1186/s12953-018-0138-4

**Published:** 2018-05-30

**Authors:** Gry H. Dihazi, Gerhard A. Mueller, Abdul R. Asif, Marwa Eltoweissy, Johannes T. Wessels, Hassan Dihazi

**Affiliations:** 1 Department of Nephrology and Rheumatology, University Medical Center Goettingen, Georg-August University Goettingen, Robert-Koch-Strasse 40, D-37075 Goettingen, Germany; 2 Department of Clinical Chemistry, Georg-August University Goettingen, Robert-Koch-Strasse 40, D-37075 Goettingen, Germany; 3 Department of Zoology, Faculty of Science, Alexandria University, Alexandria, Egypt

## Correction

Upon publication of the original article [1], Marwa Eltoweissy noticed that her affiliation: “3. Department of Zoology, Faculty of Science, Alexandria University, Alexandria, Egypt” was missing. This affiliation has now been added in this correction article.
