# Supplementary material for: Proteomic characterization of adrenal gland embryonic development reveals early initiation of steroid metabolism and reduction of the retinoic acid pathway
Source: Proteome Sci. 2015 Feb 7;13:6. doi: 10.1186/s12953-015-0063-8 (PMC4331441; doi:10.1186/s12953-015-0063-8)

Figure S1A

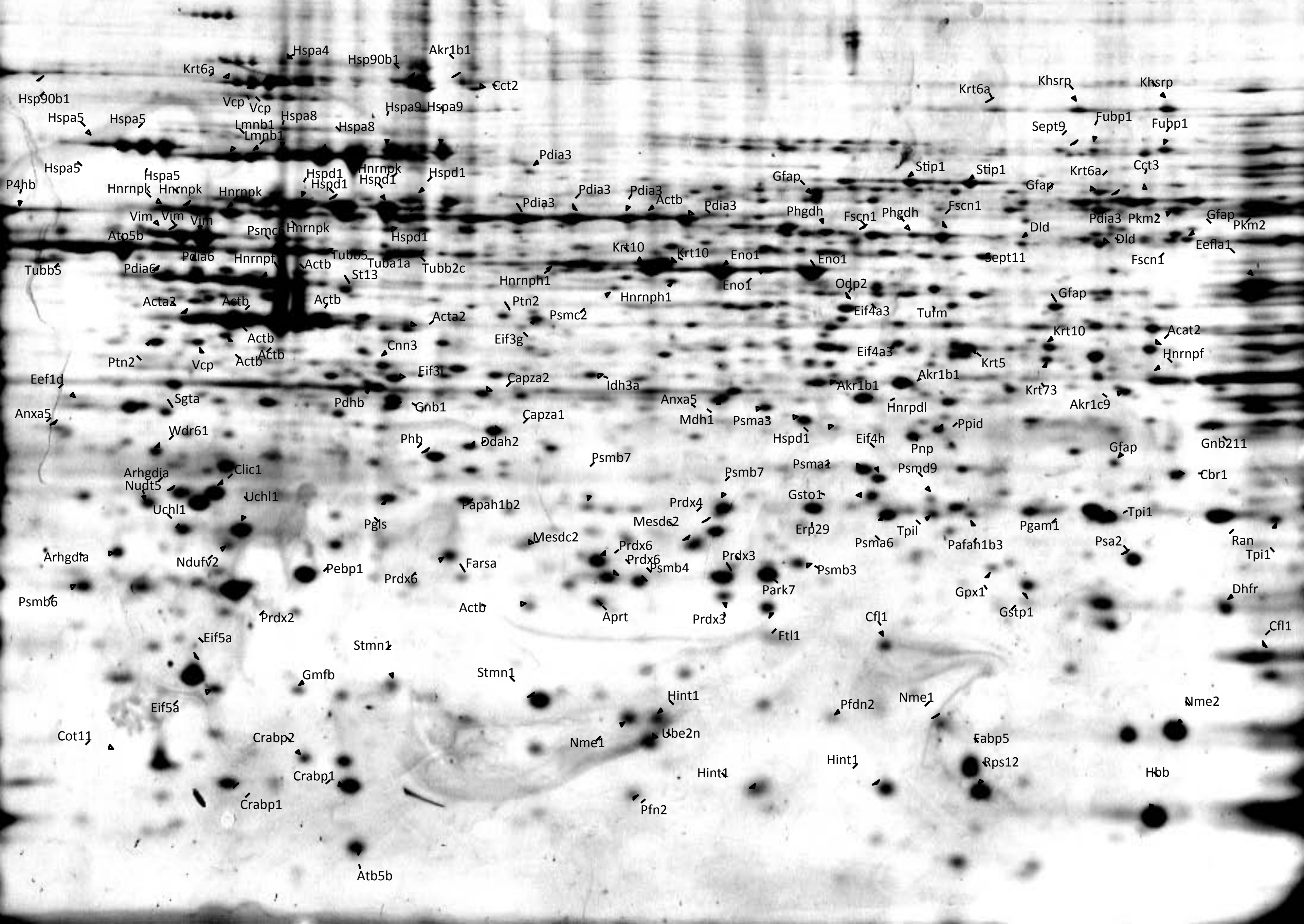

Hspa4
Hsp90b1
Akr1b1
Krt6a
Cct2
Hsp90b1
Vcp
Vcp
Hspa9
Hspa9
Krt6a
Khsrp
Khsrp
Hspa5
Hspa5
Lmnb1
Lmnb1
Hspa8
Hspa8
Sept9
Fubp1
Fubp1
Pdia3
Hspa5
Hspa5
Hnrnpk
Hspd1
Hspd1
Hspd1
Stip1
Stip1
Krt6a
Cct3
P4hb
Hnrnpk
Hnrnpk
Hnrnpk
Hspd1
Hspd1
Gfap
Gfap
Pdia3
Pdia3
Pdia3
Actb
Pdia3
Phgdh
Fscn1
Phgdh
Fscn1
Pdia3
Pkm2
Gfap
Pkm2
Vim
Vim
Vim
Psmc5
Hnrnpk
Atp5b
Hspd1
Dld
Dld
Eefla1
Krt10
Krt10
Eno1
Eno1
Sept11
Fscn1
Tubb5
Pdia6
Pdia6
Hnrnpf
Actb
Tubb5
Tuba1a
St13
Tubb2c
Hnrnph1
Eno1
Hnrnph1
Odp2
Gfap
Acta2
Actb
Actb
Ptn2
Psmc2
Acta2
Eif4a3
Tufm
Eif3g
Krt10
Acat2
Actb
Cnn3
Eif4a3
Hnrnpf
Ptn2
Vcp
Actb
Actb
Krt5
Eif3i
Eef1d
Capza2
Idh3a
Akr1b1
Akr1b1
Krt73
Sgta
Pdhb
Gnb1
Anxa5
Hnrpdl
Akr1c9
Anxa5
Capza1
Mdh1
Psma3
Ppid
Wdr61
Phb
Ddah2
Hspd1
Eif4h
Pnp
Gfap
Gnb211
Psmb7
Psma1
Psmd9
Clic1
Arhgdia
Nudt5
Psmb7
Cbr1
Uchl1
Gsto1
Prdx4
Uchl1
Papah1b2
Tpi1
Pgls
Mesdc2
Erp29
Tpil
Pgam1
Mesdc2
Psma6
Pafah1b3
Psa2
Ran
Prdx6
Arhgdia
Ndufv2
Prdx6
Prdx3
Tpi1
Farsa
Psmb4
Pebp1
Psmb3
Prdx6
Park7
Gpx1
Dhfr
Psmb6
Actb
Aprt
Gstp1
Prdx2
Prdx3
Cfl1
Cfl1
Ftl1
Eif5a
Stmn1
Gmfb
Stmn1
Hint1
Nme1
Pfdn2
Nme2
Eif5a
Cot11
Crabp2
Ube2n
Nme1
Fabp5
Hint1
Crabp1
Hint1
Rps12
Hbb
Crabp1
Pfn2
Atb5b

Figure S1B

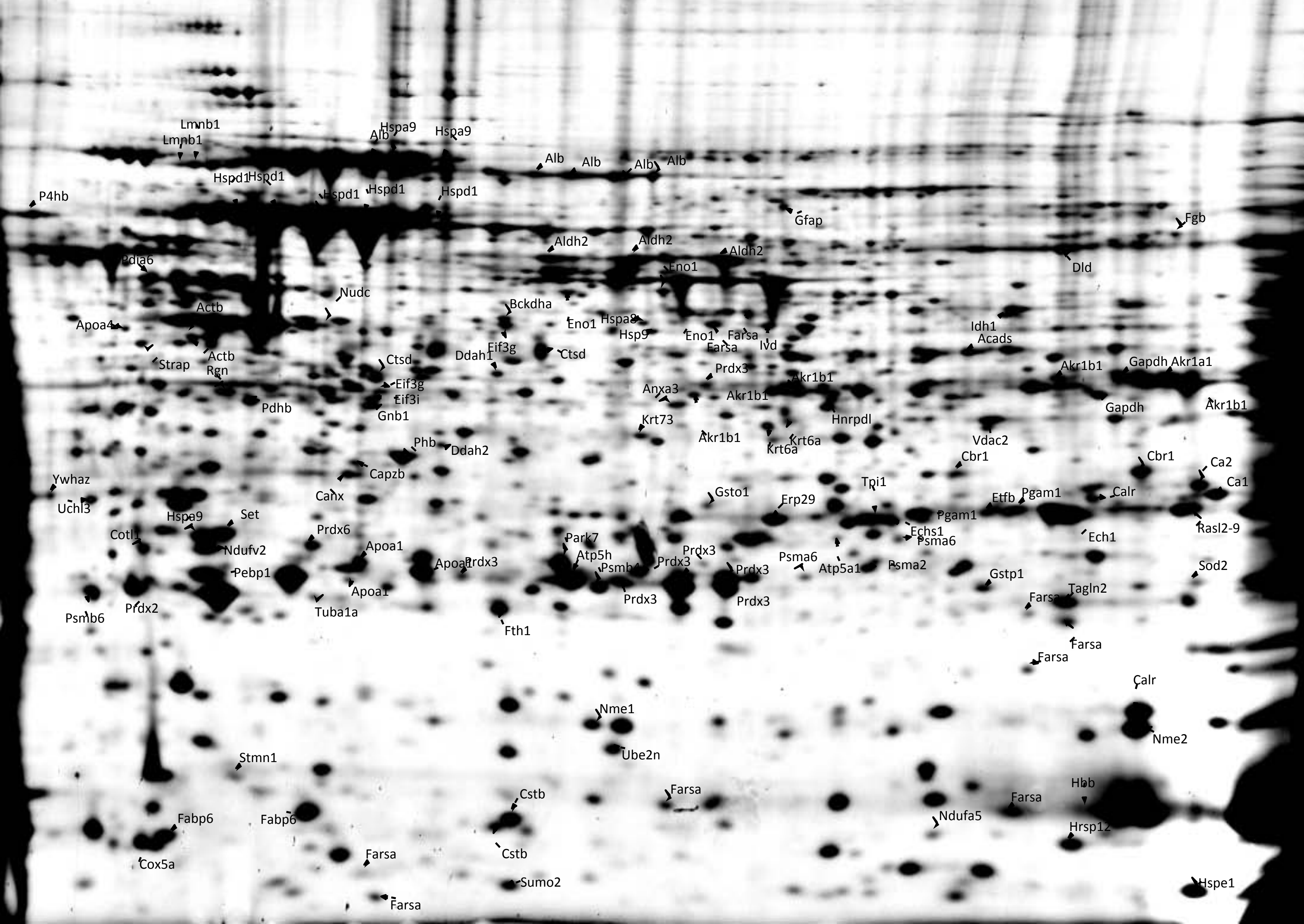

Lmnb1
Lmnb1
Hspa9
Alb
Hspa9
Alb
Alb
Alb
Alb
Hspd1
Hspd1
Hspd1
Hspd1
Hspd1
P4hb
Gfap
Fgb
Aldh2
Aldh2
Aldh2
Pdia6
Eno1
Dld
Nudc
Bckdha
Actb
Apoa4
Eno1
Hspa8
Hsp9
Eno1
Farsa
Farsa
Ivd
Idh1
Acads
Eif3g
Ctsd
Ddah1
Strap
Actb
Rgn
Ctsd
Prdx3
Eif3g
Eif3i
Akr1b1
Gapdh
Akr1a1
Akr1b1
Anxa3
Akr1b1
Pdhb
Gnb1
Gapdh
Akr1b1
Krt73
Hnrpdl
Akr1b1
Krt6a
Krt6a
Vdac2
Phb
Ddah2
Cbr1
Cbr1
Ca2
Capzb
Ywhaz
Tpi1
Ca1
Canx
Gsto1
Erp29
Etfb
Pgam1
Calr
Uchl3
Hspa9
Set
Pgam1
Cotl1
Prdx6
Park7
Echs1
Psma6
Ech1
Rasl2-9
Ndufv2
Apoa1
Atp5h
Prdx3
Psma6
Apoa1
Prdx3
Psmb4
Prdx3
Atp5a1
Psma2
Sod2
Pebp1
Prdx3
Gstp1
Apoa1
Prdx3
Tagln2
Psmb6
Prdx2
Tuba1a
Prdx3
Farsa
Fth1
Farsa
Farsa
Calr
Nme1
Nme2
Ube2n
Stmn1
Hbb
Cstb
Farsa
Farsa
Ndufa5
Fabp6
Fabp6
Hrsp12
Cox5a
Farsa
Cstb
Sumo2
Hspe1
Farsa

Figure S1C

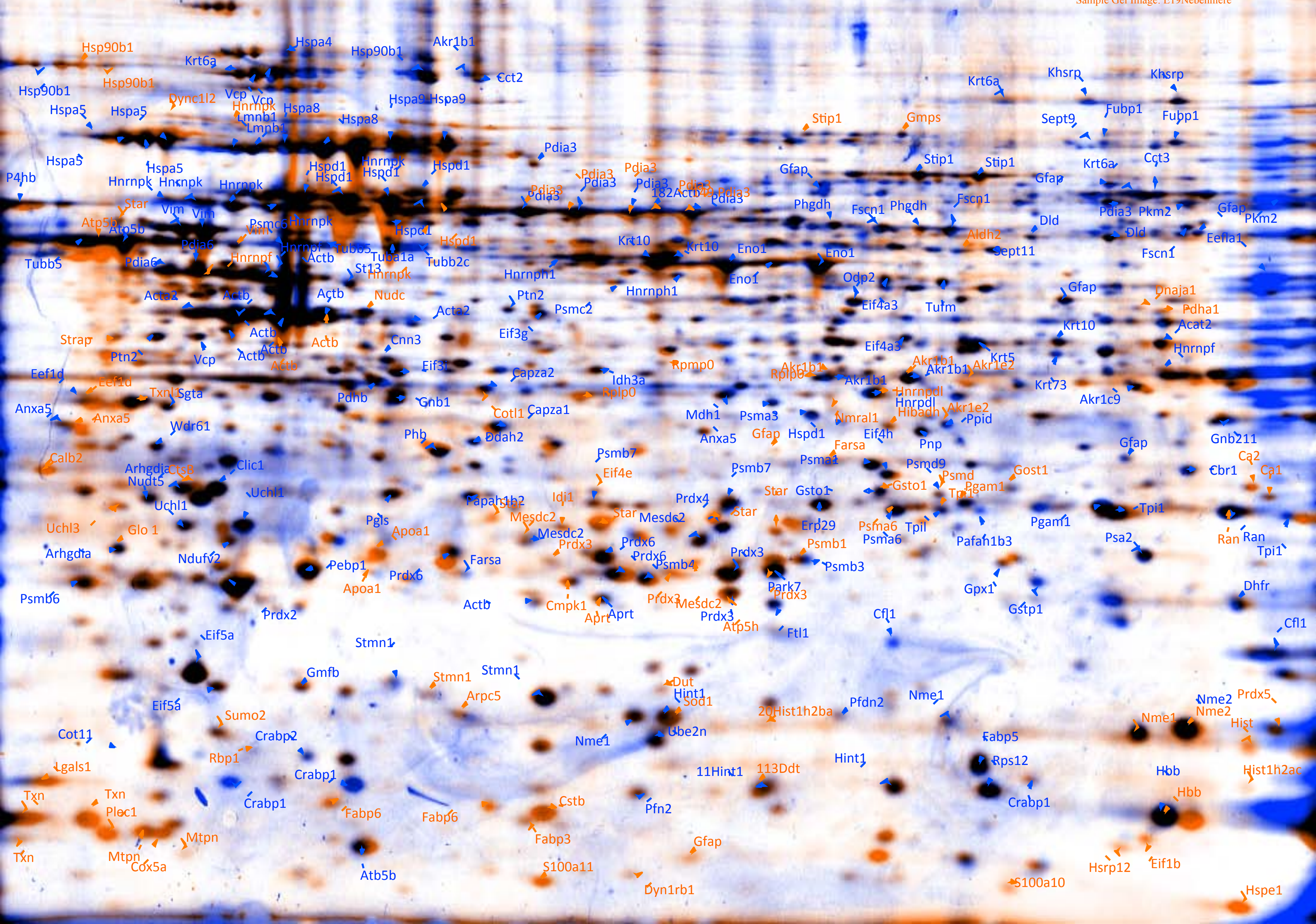

Master Gel Image: E16Nebenniere
Sample Gel Image: E19Nebenniere
Hsp90b1
Hspa4
Hsp90b1
Akr1b1
Krt6a
Cct2
Hsp90b1
Hsp90b1
Dync1i2
Vcp
Vcp
Hnrnpk
Lmnb1
Lmnb1
Hspa8
Hspa8
Hspa9
Hspa9
Hspa5
Hspa5
Krt6a
Khsrp
Khsrp
Stip1
Gmps
Sept9
Fubp1
Fubp1
Hspa5
Hspa5
P4hb
Hnrnpk
Hnrnpk
Hnrnpk
Hspd1
Hspd1
Hnrnpk
Hspd1
Hspd1
Pdia3
Pdia3
Pdia3
Pdia3
Pdia3
Pdia3
Pdia3
Pdia3
182Actb
Pdia3
Gfap
Stip1
Stip1
Krt6a
Cct3
Gfap
Star
Vim
Vim
Phgdh
Fscn1
Phgdh
Fscn1
Pdia3
Pkm2
Gfap
Pkm2
Atp5b
Atp5b
Psmc5
Hnrnpk
Vim
Hspd1
Hspd1
Dld
Dld
Aldh2
Eefla1
Pdia6
Hnrnpf
Tubb5
Tuba1a
Actb
Krt10
Krt10
Eno1
Eno1
Sept11
Fscn1
Tubb5
Pdia6
Hnrnpf
St13
Hnrnpk
Tubb2c
Hnrnph1
Eno1
Odp2
Acta2
Actb
Actb
Nudc
Ptn2
Hnrnph1
Gfap
Dnaja1
Acta2
Psmc2
Eif4a3
Tufm
Pdha1
Actb
Eif3g
Krt10
Acat2
Strap
Actb
Cnn3
Actb
Eif4a3
Ptn2
Vcp
Actb
Actb
Hnrnpf
Eif3i
Rpmp0
Akr1b1
Akr1b1
Akr1b1
Krt5
Akr1e2
Eef1d
Capza2
Rplp0
Krt73
Eef1d
Idh3a
Akr1b1
Txnl1
Sgta
Rplp0
Hnrnpdl
Akr1c9
Anxa5
Pdhb
Gnb1
Hnrpdl
Capza1
Hibadh
Akr1e2
Anxa5
Cotl1
Mdh1
Psma3
Nmral1
Ppid
Wdr61
Phb
Ddah2
Anxa5
Gfap
Hspd1
Eif4h
Pnp
Gfap
Gnb211
Calb2
Psmb7
Farsa
Ca2
Clic1
Psmb7
Psma1
Psmd9
Gost1
Arhgdia
Cts8
Eif4e
Psmd
Cbr1
Ca1
Nudt5
Uchl1
Star
Gsto1
Gsto1
Pgam1
Papah1b2
Star
Idi1
Prdx4
Tpi1
Uchl1
Star
Mesdc2
Star
Tpi1
Uchl3
Glo 1
Pgls
Mesdc2
Erp29
Psma6
Tpil
Pgam1
Ran
Ran
Apoa1
Mesdc2
Psma6
Psa2
Arhgdia
Prdx3
Prdx6
Psmb1
Pafah1b3
Tpi1
Ndufv2
Prdx6
Prdx3
Farsa
Psmb4
Psmb3
Pebp1
Prdx6
Apoa1
Park7
Gpx1
Dhfr
Psmb6
Prdx3
Prdx3
Actb
Cmpk1
Mesdc2
Gstp1
Prdx2
Aprt
Aprt
Prdx3
Cfl1
Cfl1
Atp5h
Ftl1
Eif5a
Stmn1
Gmfb
Stmn1
Stmn1
Dut
Hint1
Nme1
Sod1
Pfdn2
Nme2
Prdx5
Eif5a
Arpc5
20Hist1h2ba
Nme2
Sumo2
Nme1
Hist
Cot11
Crabp2
Ube2n
Fabp5
Nme1
Rbp1
Rps12
Lgals1
11Hint1
113Ddt
Hint1
Hbb
Hist1h2ac
Crabp1
Txn
Txn
Crabp1
Cstb
Crabp1
Hbb
Plec1
Fabp6
Fabp6
Pfn2
Mtpn
Fabp3
Gfap
Txn
Mtpn
Hsrp12
Eif1b
Cox5a
Atb5b
S100a11
S100a10
Dyn1rb1
Hspe1

Figure S1D

Master Gel Image: E16Nebenniere

Sample Gel Image: E 23Nebenniere

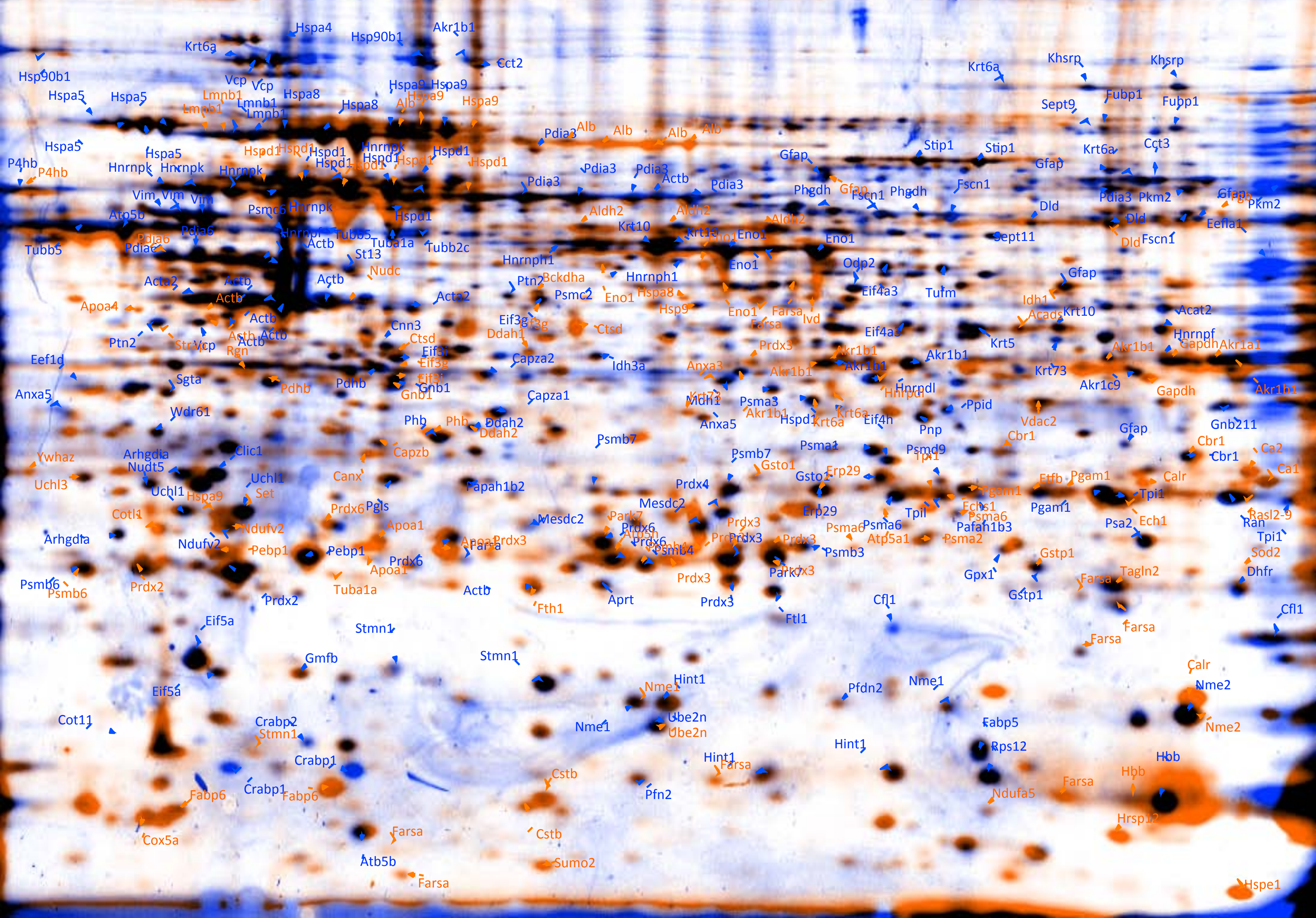

Figure S1E

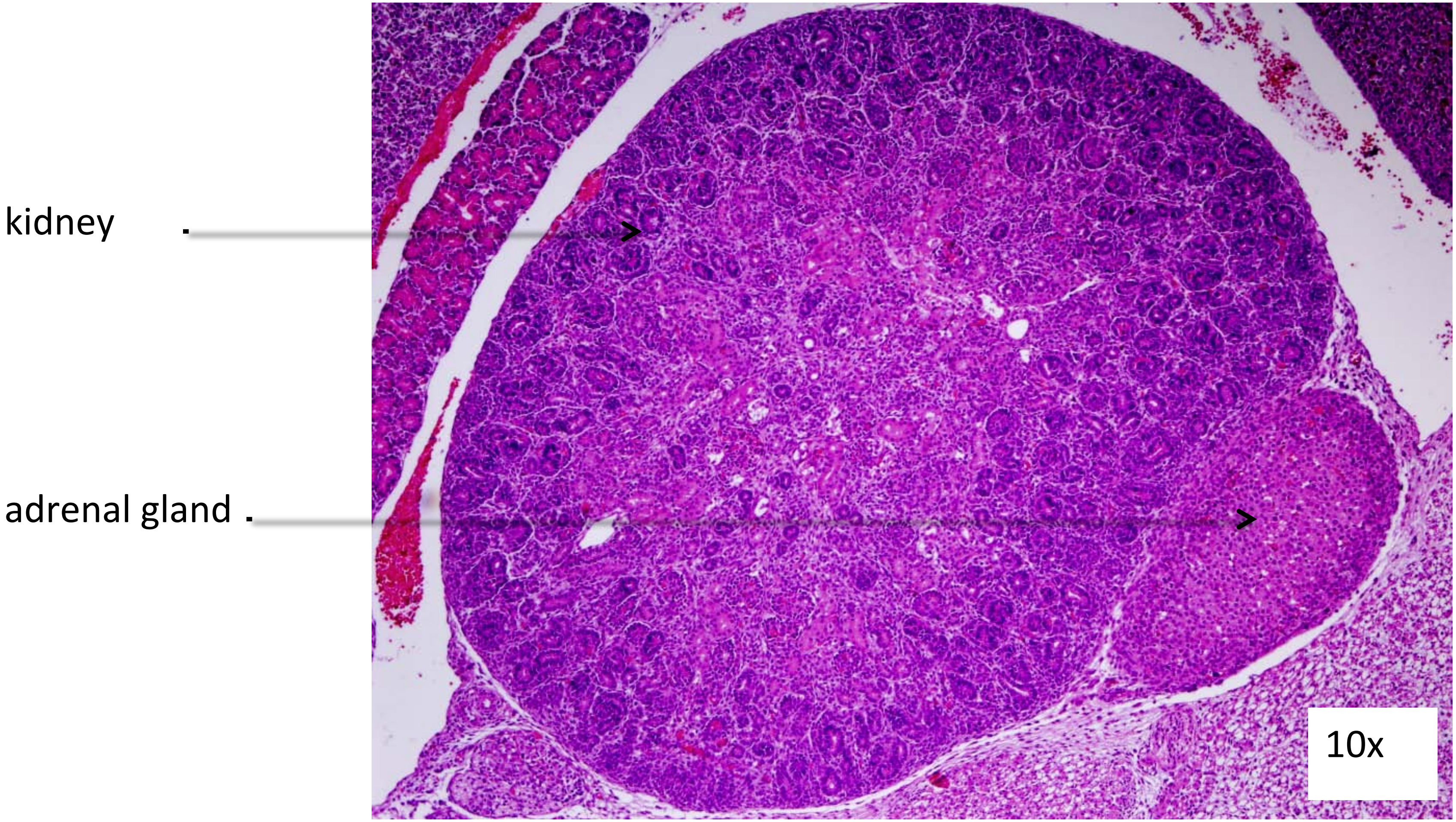

Supplement: Additional file 1: Figure S1. — A: 2D gel of the proteins in the adrenal gland of an E16 embryo and B: of a newborn rat. The identified protein spots are labeled with the gene names on the gel. C: An overlay of the 2D gels of the adrenal glands of E16 and E19 embryos. D: An overlay of the 2D gels of the adrenal glands of E16 embryo and new born. The identified spots are labeled with the gene names. E: HE staining of a paraffin section of adrenal gland and kidney at E19. [file 12953_2015_63_MOESM1_ESM.pdf]
