# Supplementary material for: Proteomic characterization of adrenal gland embryonic development reveals early initiation of steroid metabolism and reduction of the retinoic acid pathway
Source: Proteome Sci. 2015 Feb 7;13:6. doi: 10.1186/s12953-015-0063-8 (PMC4331441; doi:10.1186/s12953-015-0063-8)
Supplement: Additional file 2: Table S1. — List of proteins of non-redundant proteins identified from adrenal glands in all three analysed embryonic stages. Gene name, calculated isoelectric point (CIP) and MS/MS information are given. Additionally protein nominal mass and the number of peptides that were sequenced through MS/MS are also given. [file 12953_2015_63_MOESM2_ESM.doc]

Additional file 2 Table S1: List of proteins of non-redundant proteins identified from adrenal glands in all three analysed embryonic stages. Gene name, calculated isoelectric point (CIP) and MS/MS information are given. Additionally protein nominal mass and the number of peptides that were sequenced through MS/MS are also given.

| **Protein name** | **Nominal mass** | **CPI** | **ESI score** | **Peptides** | **Gene name** |
| --- | --- | --- | --- | --- | --- |
| short-chain specific acyl-CoA dehydrogenase, mitochondrial | 44737 | 8.5 | 363 | 10 | **Acads** |
| Acetyl COA acetyltransferase, cytosolic | 41082 | 6.9 | 105 | 5 | **Acat2** |
| Actin, cytoplasmic 1 | 41710 | 5.3 | 2574 | 11 | **Actb** |
| Alcohol dehydrogenase (NADP) | 36483 | 6.8 | 173 | 7 | **Akr1a1** |
| Aldose reductase | 35774 | 6.3 | 251 | 7 | **Akr1b1** |
| 3-alpha-hydroxysteroid dehydrogenase | 37004 | 6.7 | 133 | 3 | **Akr1c9** |
| 1,5-anhydro-D-fructose reductase | 34478 | 7.2 | 99 | 3 | **Akr1e2** |
| Serum albumin | 68686 | 6.1 | 432 | 11 | **Alb** |
| Aldehyde dehydrogenase, mitochondrial | 56453 | 6.6 | 724 | 10 | **Aldh2** |
| Annexin A3 | 36641 | 6.0 | 238 | 2 | **Anxa3** |
| Annexin A5 | 34722 | 4.9 | 417 | 8 | **Anxa5** |
| Apolipoprotein A-1 | 30043 | 5.5 | 361 | 8 | **Apoa1** |
| Apolipoprotein A-IV | 44429 | 5.1 | 42 | 3 | **Apoa4** |
| Adenine phosphoribosyltransferase | 19533 | 6.2 | 159 | 4 | **Aprt** |
| Rho GDP-dissociation inhibitor 1 | 23393 | 5.1 | 402 | 6 | **Arhgdia** |
| Actin-related protein 2/3 Complex subunit 5 | 16310 | 5.5 | 67 | 3 | **Arpc5** |
| Bifunctional purine biosynthesis protein PURH | 64168 | 6.7 | 210 | 6 | **Atic** |
| ATP synthase subunit alpha, mitochondrial | 59717 | 9.2 | 65 | 2 | **Atp5a1** |
| ATP synthase subunit beta, mitochondrial | 56318 | 5.2 | 771 | 9 | **Atp5b** |
| ATP synthase subunit d, mitochondrial | 18752 | 6.2 | 320 | 4 | **Atp5h** |
| 2-oxoisovalerate dehydrogenase subunit alpha, mitochondrial (Fragment) | 50133 | 7.7 | 56 | 2 | **Bckdha** |
| Carbonic anhydrase 1 | 28282 | 6.9 | 367 | 7 | **Ca1** |
| Carbonic anhydrase 2 | 29096 | 6.9 | 165 | 4 | **Ca2** |
| Calretinin | 31384 | 4.9 | 224 | 6 | **Calb2** |
| Calreticulin | 47966 | 4.3 | 328 | 9 | **Calr** |
| Canexin | 66236 | 4.5 | 32 | 2 | **Canx** |
| F-actin-capping protein subunit beta | 30609 | 5.7 | 164 | 10 | **Capza1** |
| F-actin-capping protein subunit alpha-2 | 32947 | 5.6 | 66 | 2 | **Capza2** |
| F-actin-capping protein subunit beta | 30609 | 5.7 | 234 | 7 | **Capzb** |
| Carbonyl reductase (NADPH) | 30559 | 8.2 | 324 | 6 | **Cbr1** |
| T-Complex protein 1 subunit beta | 57422 | 6.1 | 230 | 14 | **Cct2** |
| T-complex 1 subunit gamma | 60608 | 6.2 | 179 | 3 | **Cct3** |
| T-Complex protein 1 subunit epsilon | 59499 | 5.5 | 106 | 10 | **Cct5** |
| Cofilin 1 | 18521 | 8.3 | 221 | 5 | **Cfl1** |
| Chloride intracellular channel protein 1 | 26964 | 5.1 | 230 | 5 | **Clic1** |
| UMP-CMP kinase | 22155 | 5.7 | 263 | 3 | **Cmpk1** |
| Calponin-3 | 36412 | 5.5 | 38 | 2 | **Cnn3** |
| Coactosin-like protein | 15922 | 5.3 | 84 | 5 | **Cotl1** |
| Cytocrome C oxidase subunit 5A, mitochondrial | 16119 | 6.1 | 303 | 4 | **Cox5a** |
| Cellular retinoic acid-binding protein 1 | 15582 | 5.3 | 695 | 11 | **Crabp1** |
| Cellular retinoic acid-binding protein 2 | 15923 | 5.2 | 50 | 7 | **Crabp2** |
| Alpha-S1-casein | 24513 | 5.2 | 54 | 2 | **Csn1s1** |
| Cystatin B | 11189 | 5.9 | 132 | 4 | **Cstb** |
| Cathepsin D | 44652 | 6.7 | 298 | 7 | **Ctsd** |
| N(G), N(G)-dimethylarginine dimethylaminohydrolase 1 | 31406 | 5.8 | 304 | 7 | **Ddah1** |
| N(G),N(G)-dimethylarginine dimethylaminohydrolase 2 | 29669 | 5.7 | 359 | 11 | **Ddah2** |
| D-dopachrome decarboxylase | 13125 | 6.1 | 260 | 5 | **Ddt** |
| Dihydrofolate | 21624 | 6.8 | 46 | 2 | **Dhfr** |
| Dihydrolipoyllysine-residue acetyltransferase component of pyruvate dehydrogenase complex, mitochondrial | 67123 | 8.8 | 134 | 4 | **Dlat** |
| Dihydrolipoyl dehydrogenase, mitochondrial | 54004 | 8.0 | 323 | 9 | **Dld** |
| DnaJ homolog subfamily A member 1 | 44839 | 6.7 | 36 | 2 | **Dnaja1** |
| Deoxyuridine 5`-triophosphate nucleotidohydrolase | 21989 | 9.0 | 103 | 5 | **Dut** |
| Dynein light chain roadlock-type 1 | 10983 | 6.9 | 96 | 1 | **Dynlrb1** |
| Enoyl-CoA hydratase, mitochondrial | 31496 | 8.4 | 529 | 7 | **Echs1** |
| Elongation factor 1-delta | 31311 | 4.9 | 121 | 7 | **Eef1d** |
| Elongation factor 1-alpha 1 | 50082 | 9.1 | 14 | 7 | **Eef1a1** |
| Eukaryotic translation initiation factor 1b | 12816 | 6.8 | 109 | 2 | **Eif1b** |
| Eukaryotic translation initiation factor 3 subunit G | 35629 | 5.7 | 99 | 4 | **Eif3g** |
| Eukaryotic translation initiation factor 3 subunit I | 36438 | 5.4 | 36 | 3 | **Eif3i** |
| Eukaryotic initiation factor 4A-III | 46811 | 7.2 | 55 | 3 | **Eif4a3** |
| Eukaryotic translation initiation factor 4E | 25037 | 5.8 | 70 | 2 | **Eif4e** |
| Eukaryotic translation initiation factor 4H | 27307 | 6.7 | 64 | 1 | **Eif4h** |
| Eukaryotic translation initiation factor 5A-1 | 16821 | 5.1 | 95 | 3 | **Eif5a** |
| Alpha-enolase | 47098 | 6.2 | 1482 | 14 | **Eno1** |
| Endoplasmic reticulum resident protein 29 | 28557 | 6.2 | 339 | 8 | **Erp29** |
| Electron transfer flavoprotein beta | 27670 | 7.6 | 95 | 6 | **Etfb** |
| Fatty acid-binding protein, heart | 14766 | 5.9 | 41 | 3 | **Fabp3** |
| Fatty acid-binding protein, epideral | 15050 | 7.0 | 978 | 8 | **Fabp5** |
| Gastrotropin | 14477 | 5.5 | 150 | 2 | **Fabp6** |
| Fibrinogen beta chain | 54201 | 7.9 | 42 | 4 | **Fgb** |
| Fascin | 54457 | 6.3 | 357 | 7 | **Fscn1** |
| Ferritin heavy chain | 21113 | 5.9 | 95 | 5 | **Fth1** |
| Ferritin light chain 1 | 20736 | 6.0 | 339 | 6 | **Ftl1** |
| FAR upstream element-binding protein 1 | 67155 | 7.3 | 122 | 3 | **Fubp1** |
| Glyceraldehyde-3-phosphate dehydrogenase | 35805 | 8.1 | 54 | 4 | **Gapdh** |
| Glial fibrillary acidic protein | 49927 | 5.4 | 67 | 2 | **Gfap** |
| Lactoylglutathione lysase | 20806 | 5.1 | 27 | 3 | **Glo1** |
| Glia maturation factor beta | 16726 | 5.3 | 36 | 2 | **Gmfb** |
| GMP synthase (glutamine-hydrolyzing) | 76709 | 6.2 | 107 | 5 | **Gmps** |
| Guanine nucleotide-binding protein G(I)/G(S)/G(T) subunit beta-1 | 37353 | 5.6 | 91 | 3 | **Gnb1** |
| Guanine nucleotide-binding protein G(I) G(S)/G(T) subunit beta-2 | 37307 | 5.6 | 149 | 4 | **Gnb2** |
| Guanine nucleotide-binding protein subunit beta-2-likiie 1 | 35055 | 7.6 | 65 | 3 | **Gnb2l1** |
| Glutathione peroxidase 1 | 22292 | 7.7 | 30 | 2 | **Gpx1** |
| GrpE protein homolog 1, mitochondrial | 24282 | 8.6 | 23 | 5 | **Grpel1** |
| Glutathione S-transferase omega-1 | 27651 | 6.3 | 168 | 8 | **Gsto1** |
| Glutathione S-transferase P | 23424 | 6.9 | 322 | 5 | **Gstp1** |
| Hemoglobin subunit beta-1 | 15969 | 7.9 | 188 | 4 | **Hbb** |
| 3-hydroxyisobutyrate dehydrogenase, mitochondrial | 35280 | 8.7 | 447 | 6 | **Hibadh** |
| Histidine triad nucleotide-binding protein1 | 13768 | 6.4 | 134 | 4 | **Hint1** |
| Histone H2A type 1-C | 14097 | 11.1 | 104 | 3 | **Hist1h2ac** |
| Histone H2B type 1-A | 14216 | 10.3 | 71 | 2 | **Hist1h2ba** |
| Heterogeneous nuclear ribonucleoprotein F | 45701 | 5.3 | 115 | 4 | **Hnrnpf** |
| Heterogeneous nuclear ribonucleprotein H | 49157 | 5.7 | 176 | 5 | **Hnrnph1** |
| Heterogeneous nuclear ribonucleoprotein K | 50944 | 5.4 | 583 | 9 | **Hnrnpk** |
| Heterogeneous nuslear ribonucleoprotein D-like | 35272 | 9.1 | 264 | 4 | **Hnrpdl** |
| Endoplasmin | 92713 | 4.7 | 237 | 10 | **Hsp90b1** |
| Heat shock 70 kDa protein 4 | 93997 | 5.1 | 45 | 3 | **Hspa4** |
| 78 kDa glucose-regulated protein | 72302 | 5.0 | 932 | 14 | **Hspa5** |
| Heat shock cognate 71 kDa protein | 70827 | 5.4 | 312 | 10 | **Hspa8** |
| Stress-70 protein, mitochondrial | 73812 | 5.5 | 1240 | 16 | **Hspa9** |
| Heat shock protein beta-1 | 24822 | 6.1 | 56 | 4 | **Hspb1** |
| 60 kDa heat shock protein, mitochondrial | 60917 | 5.4 | 1675 | 21 | **Hspd1** |
| 10 kDa heat shock protein, mitochondrial | 10895 | 8.9 | 567 | 7 | **Hspe1** |
| Ribinuclease UK114 | 14295 | 7.8 | 134 | 4 | **Hrsp12** |
| Isocitrate dehydrogenase ( NADP ) cytoplasmic | 46705 | 6.5 | 290 | 11 | **Idh1** |
| Isocitrate dehydrogenase (NAD) subunit alpha, mitrochondrial | 39588 | 6.5 | 176 | 7 | **Idh3a** |
| Isopentenyl-diphosphate Delte-isomerase 1 | 26379 | 5.6 | 126 | 4 | **Idi1** |
| Isovaleryl-CoA dehydrogenase, mitochondrial | 46406 | 8.0 | 271 | 6 | **Ivd** |
| Far upstream element-binding protein 2 | 74180 | 6.4 | 151 | 6 | **Khsrp** |
| Keratin, type II cytoskeletal 1 | 64791 | 8.0 | 193 | 3 | **Krt1** |
| Keratin, type I cytoskeletal 10 | 56470 | 5.1 | 204 | 8 | **Krt10** |
| Keratin, type 1 Cytoskeletal 15 | 48840 | 4.7 | 87 | 2 | **Krt15** |
| Keratin, type II cytoskeletal 5 | 61788 | 7.6 | 48 | 2 | **Krt5** |
| Keratin, type II Cytoskeletal 6A | 59213 | 8.1 | 203 | 2 | **Krt6a** |
| Keratin, type II cytoskeletal 73 | 58875 | 8.2 | 195 | 3 | **Krt73** |
| Galectin-1 | 14847 | 5.1 | 260 | 5 | **Lgals1** |
| Lamin-B1 | 66566 | 5.2 | 332 | 11 | **Lmnb1** |
| Malate dehydrogenase, cytoplasmic | 36460 | 6.2 | 166 | 4 | **Mdh1** |
| LDLR chaperone MESD | 25200 | 5.5 | 139 | 4 | **Mesdc2** |
| Myotrophin | 12853 | 5.3 | 97 | 2 | **Mtpn** |
| NADH dehydrogenase (ubiquinone) 1 alpha subcomplex subunit 5 | 13403 | 6.8 | 55 | 3 | **Ndufa5** |
| NADH dehydrogenase (ubiquinone) flavoprotein 2, mitochondrial | 27361 | 6.2 | 184 | 3 | **Ndufv2** |
| Nucleoside diphosphate kinase A | 17182 | 6.0 | 263 | 5 | **Nme1** |
| Nucleoside diphosphate kinase B | 17272 | 7.1 | 920 | 10 | **Nme2** |
| NmrA-like family domain-containing protein 1 | 16891 | 7.1 | 53 | 3 | **NmraI1** |
| Nuclear migration protein nudC | 38388 | 5.3 | 98 | 7 | **Nudc** |
| ADP-sugar pyropgoshhhhpatease | 24102 | 5.1 | 421 | 7 | **Nudt5** |
| Protein disulfide-isomerase | 56916 | 4.8 | 543 | 10 | **P4hb** |
| Platelet-activating factor acetylhygrolase IB subunit beta | 25565 | 5.6 | 110 | 3 | **Pafah1b2** |
| Platelet-activating factor acetylhygrolase IB subunit gamma | 25847 | 6.4 | 112 | 2 | **Pafah1b3** |
| Protein DJ-1 | 19961 | 6.3 | 140 | 7 | **Park7** |
| Pyruvate dehydrogenaseE1 component subunit alpha, | 43199 | 8.5 | 72 | 3 | **Pdha1** |
| Pyruvate dehydrogenase E1 Complement subunit beta, mitrochondrial | 38957 | 6.2 | 318 | 7 | **Pdhb** |
| Protein disulfide-isomerase A3 | 56588 | 5.9 | 856 | 15 | **Pdia3** |
| Protein disulfide-isomerase A6 | 48143 | 5.0 | 598 | 8 | **Pdia6** |
| Phosphatidylethanolamine-binding protein1 | 20788 | 5.5 | 398 | 4 | **Pebp1** |
| Prefoldin subunit 2 | 16570 | 6.2 | 183 | 3 | **Pfdn2** |
| Profilin-2 | 14992 | 6.8 | 195 | 2 | **Pfn2** |
| Phosphoglycerate mutase 1 | 28814 | 6.8 | 187 | 8 | **Pgam1** |
| 6-phosphogluconolactonase | 27217 | 5.5 | 363 | 7 | **Pgls** |
| Prohibitin | 29802 | 5.6 | 775 | 8 | **Phb** |
| D-3-phosphoglycerate dehydrogenase | 56457 | 6.3 | 79 | 3 | **Phgdh** |
| Pyruvate kinase isozymes M1M2 | 57781 | 6.6 | 156 | 7 | **Pkm2** |
| Plectin-1 | 533214 | 5.7 | 26 | 2 | **Plec1** |
| Purine nucleoside phosphorylase | 32281 | 6.5 | 58 | 5 | **Pnp** |
| Peptidyl-prolyl cis-trans isomerase D | 40740 | 6.7 | 57 | 2 | **Ppid** |
| Peroxiredoxin-2 | 21770 | 5.3 | 321 | 4 | **Prdx2** |
| Thioredoxin- dependent peroxide reductase, mitochondrial | 28277 | 7.1 | 267 | 7 | **Prdx3** |
| Peroxiredoxin-4 | 30988 | 6.2 | 103 | 3 | **Prdx4** |
| Peroxiredoxin-5, mitochondrial | 22165 | 8.9 | 454 | 6 | **Prdx5** |
| Peroxiredoxin-6 | 24803 | 5.6 | 251 | 9 | **Prdx6** |
| Proteasome subnit alpha type-1 | 29499 | 6.2 | 226 | 10 | **Psma1** |
| Proteasome subnit alpha type-2 | 25910 | 6.9 | 197 | 6 | **Psma2** |
| Proteasome subunit alpha type-3 | 28401 | 5.3 | 42 | 1 | **Psma3** |
| Proteasome subunit alpha type-6 | 27382 | 6.3 | 336 | 5 | **Psma6** |
| Proteasome subunit beta type-3 | 22949 | 6.2 | 216 | 2 | **Psmb3** |
| Proteasome subunit beta type-4 | 29178 | 6.5 | 179 | 3 | **Psmb4** |
| Proteasome subunit beta type-6 | 25273 | 4.8 | 289 | 3 | **Psmb6** |
| Proteasome subnit beta type 7 | 29908 | 8.1 | 66 | 2 | **Psmb7** |
| 26S protease regulatory subnit 7 | 48544 | 5.6 | 35 | 2 | **Psmc2** |
| 26S protease regulatory subnit 6A | 49129 | 5.1 | 231 | 7 | **Psmc6** |
| 26S proteasome non-ATPase regulatory subunit 9 | 24814 | 6.4 | 97 | 3 | **Psmd9** |
| Proteasome activator complex subnit 1 | 28559 | 5.8 | 58 | 6 | **Psme1** |
| GTP-binding nuclear protein Ran | 24408 | 7.2 | 282 | 9 | **Ran** |
| GTP-binding nuclear protein Ran, testis-specific isoform | 24436 | 6.6 | 193 | 3 | **Rasl2-9** |
| Retinol-binding Protein 1 | 15824 | 5.1 | 52 | 2 | **Rbp1** |
| Regucalcin | 33368 | 5.3 | 214 | 4 | **Rgn** |
| 6OS acidic ribosamal protein PO | 34194 | 5.9 | 398 | 7 | **Rplp0** |
| 40S ribosomal protein S1 2 | 14516 | 7.0 | 102 | 4 | **Rps12** |
| Protein S100-A10 | 11068 | 6.3 | 42 | 2 | **S100a10** |
| Protein S100-A11 | 11057 | 5.6 | 217 | 1 | **S100a11** |
| Septin-11 | 49663 | 6.2 | 228 | 7 | **Sept11** |
| Septin-9 | 63752 | 8.7 | 33 | 2 | **Sept9** |
| Protein SET | 33386 | 4.2 | 110 | 4 | **Set** |
| Small glutamie-rich tetratricopeptide repeat-containing protein alpha | 34136 | 5.1 | 41 | 2 | **Sgta** |
| Superoxide dismutase | 15902 | 5.9 | 286 | 4 | **Sod1** |
| Superoxide dismutase, mitochondrial | 24588 | 9.0 | 34 | 4 | **Sod2** |
| Hsc70-interacting protein | 41253 | 5.3 | 79 | 3 | **St13** |
| Steroidogenic acute regulatory protein,mitochondrial | 31481 | 6.0 | 856 | 6 | **Star** |
| Stress-induced-phosphoprotein1 | 62530 | 6.4 | 246 | 15 | **Stip1** |
| Stathmin | 17278 | 5.8 | 190 | 3 | **Stmn1** |
| Serine-threonine kinase receptor-associated protein | 38432 | 5.0 | 195 | 6 | **Strap** |
| Suppressor of G2 allele of SKP1 homolog | 38067 | 5.1 | 92 | 3 | **Sugt1** |
| Small ubiquitin-related modifier 2 | 10864 | 5.3 | 123 | 2 | **Sumo2** |
| Transgelin-2 | 22381 | 8.4 | 175 | 2 | **Tagln2** |
| Triosephosphate isomerase | 26832 | 7.1 | 375 | 11 | **Tpi1** |
| Tubulin beta-5 chain | 49639 | 4.8 | 243 | 8 | **Tuba1a** |
| Tubulin beta-2C chain | 49769 | 4.8 | 218 | 6 | **Tubb2c** |
| Tubulin beta-5 chain | 49639 | 4.8 | 444 | 13 | **Tubb5** |
| Elongation factor Tu, mitochondrial | 49491 | 6.2 | 111 | 5 | **Tufm** |
| Thioredoxin | 18220 | 5.8 | 327 | 3 | **Txn** |
| Thioredoxin-like protein 1 | 32229 | 4.8 | 109 | 3 | **Txnl1** |
| Ubiquitin-conjugating enzyme E2N | 17113 | 6.2 | 162 | 6 | **Ube2n** |
| Ubiquitin-carboxyl-terminal hydrolase isozyme L1 | 24822 | 5.1 | 61 | 4 | **Uchl1** |
| Ubiquitin carboxyl-terminal hydrolase isozyme L3 | 26107 | 5.0 | 167 | 5 | **Uchl3** |
| Transitional endoplasmic reticulum ATPase | 89293 | 5.1 | 467 | 16 | **Vcp** |
| Voltage-dependent anion-selective channel ptotein 2 | 31726 | 7.4 | 252 | 4 | **Vdac2** |
| Vimentin | 53700 | 5.1 | 772 | 18 | **Vim** |
| WD repeat-containing protein 61 | 33726 | 5.2 | 52 | 2 | **Wdr61** |
| 14-3-3 protein theta | 27761 | 4.7 | 37 | 4 | **Ywhaq** |
| 14-3-3 protein zeta/delta | 27754 | 4.7 | 37 | 3 | **Ywhaz** |
| Histone H2B type 1 | 13982 | 10.4 | 198 | 3 | **Hist1h2ba** |
