# Supplementary figures and images for: Proteomic characterization of adrenal gland embryonic development reveals early initiation of steroid metabolism and reduction of the retinoic acid pathway
Source: Proteome Sci. 2015 Feb 7;13:6. doi: 10.1186/s12953-015-0063-8 (PMC4331441; doi:10.1186/s12953-015-0063-8)

## Slide 1
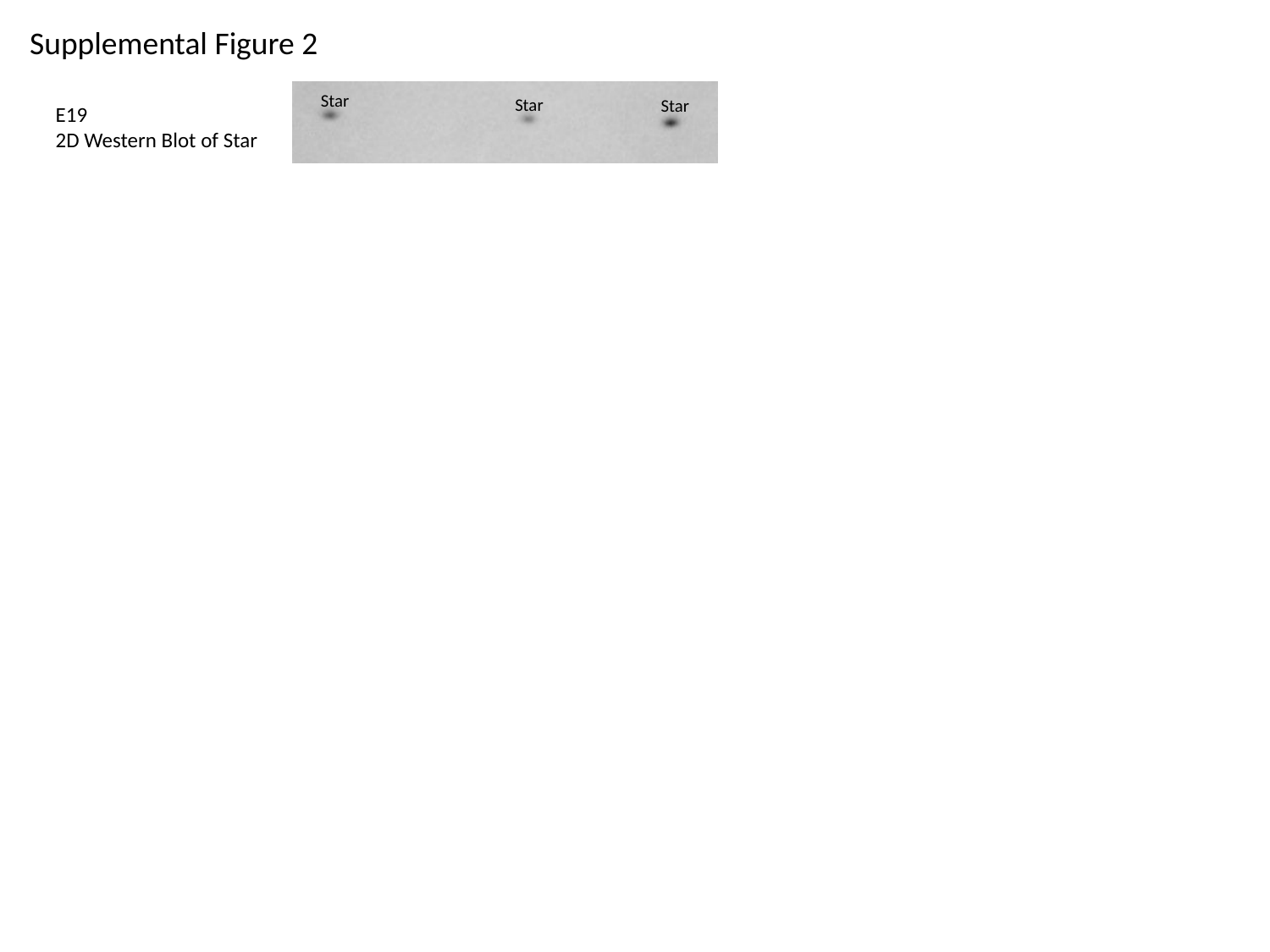

Supplemental Figure 2
E19
2D Western Blot of Star
Star
Star
Star

Supplement: Additional file 3: Figure S2. — 2D Western blot of Star in the adrenal gland at E19. Three different spots with the same molecular mass, but with different pI were observed. [file 12953_2015_63_MOESM3_ESM.ppt]
